# Adaptive Immune Neuroprotection in G93A-SOD1 Amyotrophic Lateral Sclerosis Mice

**DOI:** 10.1371/journal.pone.0002740

**Published:** 2008-07-23

**Authors:** Rebecca Banerjee, R. Lee Mosley, Ashley D. Reynolds, Alok Dhar, Vernice Jackson-Lewis, Paul H. Gordon, Serge Przedborski, Howard E. Gendelman

**Affiliations:** 1 Department of Pharmacology and Experimental Neuroscience, Center for Neurovirology and Neurodegenerative Disorders, University of Nebraska Medical Center, Omaha, Nebraska, United States of America; 2 Department of Neurology, Center for Motor Neuron Biology and Disease, Eleanor and Lou Gehrig MDA/ALS Research Center, Columbia University, New York, New York, United States of America; Columbia University, United States of America

## Abstract

**Background:**

Innate neuroimmune dysfunction is a pathobiological feature of amyotrophic lateral sclerosis (ALS). However, links, if any, between disease and adaptive immunity are poorly understood. Thus, the role of T cell immunity in disease was investigated in human G93A superoxide dismutase 1 (SOD1) transgenic (Tg) mice and subsequently in ALS patients.

**Methods and Findings:**

Quantitative and qualitative immune deficits in lymphoid cell and T cell function were seen in G93A-SOD1 Tg mice. Spleens of Tg animals showed reductions in size, weight, lymphocyte numbers, and morphological deficits at terminal stages of disease compared to their wild-type (Wt) littermates. Spleen sizes and weights of pre-symptomatic Tg mice were unchanged, but deficits were readily seen in T cell proliferation coincident with increased annexin-V associated apoptosis and necrosis of lymphocytes. These lymphoid deficits paralleled failure of Copolymer-1 (COP-1) immunization to affect longevity. In addition, among CD4^+^ T cells in ALS patients, levels of CD45RA^+^ (naïve) T cells were diminished, while CD45RO^+^ (memory) T cells were increased compared to age-matched caregivers. In attempts to correct mutant SOD1 associated immune deficits, we reconstituted SOD1 Tg mice with unfractionated naïve lymphocytes or anti-CD3 activated CD4^+^CD25^+^ T regulatory cells (Treg) or CD4^+^CD25^−^ T effector cells (Teff) from Wt donor mice. While naive lymphocytes failed to enhance survival, both polyclonal-activated Treg and Teff subsets delayed loss of motor function and extended survival; however, only Treg delayed neurological symptom onset, whereas Teff increased latency between disease onset and entry into late stage.

**Conclusions:**

A profound and progressive immunodeficiency is operative in G93A-SOD1 mice and is linked to T cell dysfunction and the failure to elicit COP-1 neuroprotective immune responses. In preliminary studies T cell deficits were also observed in human ALS. These findings, taken together, suggest caution in ascribing vaccination outcomes when these animal models of human ALS are used for study. Nonetheless, the abilities to improve neurological function and life expectancy in G93A-SOD1 Tg mice by reconstitution with activated T cells do provide opportunities for therapeutic intervention.

## Introduction

Innate immune dysfunction is a pathogenic feature of amyotrophic lateral sclerosis (ALS) [1], [2]. Transgenic (Tg) mice overexpressing mutated human G93A superoxide dismutase 1 (SOD1) [3] recapitulate ALS pathobiology including neuroinflammatory responses and motor neuron degeneration [4]–[7]. Microglial inflammatory responses contribute to progressive neuronal loss in SOD1 mutant Tg mice and in human ALS [8]–[12]. Functional ties between adaptive immunity and neurodegenerative disease are known for Parkinson's disease [13], [14], Alzheimer's disease (AD) [15]–[17], and multiple sclerosis (MS) [18], [19]. Moreover, neuroprotective responses by Copolymer-1 (COP-1) immunization were observed in animal models of these and other neurodegenerative disorders [20]–[30]. However, links between adaptive immunity and ALS remains obscure. Changes in T cell numbers and adaptive immune molecules in postmortem ALS and SOD1 Tg mouse nervous system tissues were reported [6], [31]–[36]. Interestingly, such COP-1 immunization strategies yielded mixed results in G93A-SOD1 mice [37]–[39]. Taken together, these findings suggest a progressive immune dysfunction in G93A-SOD1 mice.

Mutant SOD1 may play a role in progression of ALS as microglia recovered from G93A-SOD1 mice induce increased motoneuron injury than microglia from over-expressing wild-type (Wt) human SOD1 [8], [40]. Human ALS immunocytes show that both activated monocytes and T cell numbers are linked to disease progression [41], [42]. These data are consistent with a disease model where systemic immunologic activation plays an active role in ALS progression [31], [40], [41], [43].

Based on these observations, we investigated T cell phenotype and function in G93A-SOD1 Tg mice and in ALS patients. In support of our own observations and those made by others [38], [39] COP-1 immunization provided clinical benefit to only female G93A-SOD1 Tg mice. We observed profound T cell functional deficits in pre-symptomatic male G93A-SOD1 Tg mice spleen as well as acute lymphopenia in end stage animals. Transfer of naïve lymphoid cells from Wt donor mice to SOD1 Tg recipient mice failed to affect survival or overcome the observed lymphopenia. As COP-1 is linked to neuroprotective T regulatory cells (Treg) and the modulation of neuroinflammatory responses [23], [28], [44], we next investigated whether CD4^+^CD25^+^ Treg or CD4^+^CD25^−^ T effector cells (Teff) could affect neurological deficits and survival. Importantly, for SOD1 Tg mice, polyclonal-activated Wt Treg or Teff administered by adoptive transfer extended longevity and attenuated motor deficits. Treg delayed clinical symptom onset, while Teff increased latency from onset to late stage disease. These results together with supportive data in human ALS suggest the presence of aberrant T cell subsets in disease.

## Methods

### Animals

Mice from two SOD1 Tg mouse strains expressing the G93A mutation, B6SJL-TgN(SOD1*G93A)1Gur (stock number, 002726; hereafter designated B6SJL SOD1 Tg) and B6.Cg-Tg(SOD1*G93A)1Gur/J (stock number, 004435; hereafter designated B6 SOD1 Tg) [4], and age- and sex-matched Wt littermates were obtained from Jackson Laboratory (Bar Harbor, ME). B6SJL SOD1 Tg mice survive from 16–20 weeks, while B6 SOD1 Tg mice have a delayed survival phenotype of 19–22 weeks. Mice were randomly separated to control and treatment groups upon receipt. All animal procedures met with National Institutes of Health guidelines and were approved by the Institutional Animal Care and Use Committee (IACUC) of the University of Nebraska Medical Center.

### Human Subjects

Experimental procedures involving human subjects were conducted in conformance with the policies and principles contained in the Federal Policy for the Protection of Human Subjects (U.S. Office of Science and Technology Policy) and in the Declaration of Helsinki.

### COP-1 Immunization

B6 SOD1 Tg mice (7 weeks old) were immunized with 75 µg of COP-1 in 0.1 ml PBS weekly (q1wk) or every 2 weeks (q2wk), or treated with PBS alone. Subcutaneous injections were administered in the flanks with a 50 µl bolus given to each side.

### Spleen Morphology, Weight, Viable Cell Counts

Spleens from Wt and Tg mice were measured and weighed. Single cell suspensions were prepared by pressing spleens through 60 µm sterile wire mesh screens in Hanks' balanced salt solution (HBSS, Mediatech Inc., Herndon, VA). Erythrocytes were lysed with ammonium chloride potassium buffer and leukocytes washed by centrifugation. Numbers of viable splenic leukocytes were determined by trypan blue exclusion of hemocytometer counts.

### Lymphocyte Proliferation

Splenocytes from individual animals were plated in 96-well round-bottom plates at 1×10^6^ cell/ml in RPMI medium 1640 (Gibco, Carlsbad, CA) supplemented with 10% fetal bovine serum, 2 mM L-glutamine, 25 mM HEPES, 1 mM sodium pyruvate, 1× nonessential amino acids, 55 mM 2-mercaptoethanol, 100 units/ml penicillin, and 100 µg/ml streptomycin (complete RPMI 1640) (Mediatech Inc.). Quadruplicate replicates were stimulated with anti-CD3 (1 µg/ml) (clone 145-2C11, BD Pharmingen), goat anti-IgM (20 µg/ml) (Jackson Immuno Research, West Grove, PA) or cultured in media alone at 37°C in 5% CO_2_ for 3 days. From Tg mice immunized with COP-1 (Sigma-Aldrich, St. Louis, MO), spleen cells were cultured in the presence of COP-1 (5 µg/ml), concanavalin A (Con A, 2 µg/ml, Sigma-Aldrich), or media for 5 days. Cells were pulsed for the final 18 hrs of incubation with 1 µCi [^3^H] methylated thymidine ([^3^H]-TdR) (MP Biomedicals ICN, Solon, OH), harvested onto glass-fiber filters, and counted by β-scintillation spectrometry (Top Count, Packard Instrument Co., Meriden, CT). Levels of spleen cell proliferation for each animal were normalized to levels of proliferation obtained from cells cultured in media alone and were reported as a stimulation index.

### Immunohistochemical Assays

Fresh frozen spleens of Tg mice and Wt littermates were embedded in OCT media (Sakura Fintek, Torrance, CA) and sectioned at 10 µm using a cryostat (CM1900, Leica, Bannockburn, IL). Sections were collected on slides and fixed in ice-cold acetone-methanol (1∶1) for 30 min. Slides were washed in phosphate-buffered saline (PBS) at room temperature (RT) and quenched for endogenous peroxidase activity in 3% hydrogen peroxide in methanol for 15 min. Nonspecific staining was blocked with 5% normal rabbit serum (NRS) (Vector Laboratories, Burlingame, CA) in PBS for 1 hr. For immunostaining, primary antibodies (clone designations and dilutions) included anti-CD3 (clone 17A2, 1∶100), anti-CD19 (clone 1D3, 1∶100), anti-F4/80 (clone BM8, 1∶500) and anti-Gr-1 (clone RB6-8C5, 1∶100) (all obtained from eBioscience, San Diego, CA). Sections were incubated with primary antibody diluted in PBS/5% NRS for 90 min at RT, washed in PBS and incubated with polyclonal rabbit anti-rat immunoglobulin (1∶400) (Dako, Capinteria, CA) for 30 min followed by streptavidin-horseradish peroxidase solution (ABC Elite vector kit, Vector Laboratories) for 30 min. Staining was visualized by addition of hydrogen peroxide substrate and diaminobenzidine chromogen (DAB substrate kit for peroxidase, Vector Laboratories) solution. Sections were counterstained with hematoxylin (Surgipath Medical Industries, Inc., Richmond, IL), dehydrated, covered with mounting media (Cytoseal 60, Kalamazoo, MI) and mounted with a glass coverslip. Slides were examined under a light microscope (Eclipse E800, Nikon, Inc., Melville, NY) and representative images captured at 100× magnification. Follicle counts, area per follicle, and densities of CD3, CD19, F4/80 and Gr-1 expression were evaluated from 4 fields/animal by digital image analysis using Image-Pro Plus version 4 software (Media Cybernetics, Silver Spring, MD).

### Flow Cytometric (FCM) Analysis of Mouse and Human Leukocytes

Single cell suspensions of spleens from Wt and Tg mice were stained with fluorescein isothiocyanate (FITC)-conjugated (clone designate) anti-CD19 (1D3), anti-CD4 (RM4-4), anti-CD62L (Mel-14), and anti-Gr-1 (RB6-8C5); phycoerythrin (PE)-conjugated (clone) anti-CD4 (GK1.5), anti-CD8b (53-5.8), and F4/80 (BM8, eBioscience); and allophycocyanin (APC)-conjugated (clone) anti-CD3 (145-2C11) and anti-CD44 (1M7). All antibodies except where indicated were obtained from BD Pharmingen (San Diego, CA).

Peripheral blood from 10 ALS patients and their age-matched caregivers were collected in ethylenediaminetetraacetic acid (EDTA) containing glass tubes at Columbia University, shipped overnight, and processed upon arrival at the University Nebraska Medical Center. Complete blood count (CBC) and differential analysis for each donor and patient were determined from samples obtained prior to shipping. For FCM analysis, 20 µl of appropriate fluorochrome-conjugated antibodies were added to 100 µl of whole blood and incubated in the dark for 30 min at RT. Erythrocytes were lysed and leukocytes fixed with FACS Lysing solution (BD Biosciences). Antibodies (clone) utilized in these studies included FITC-conjugated anti-CD8a (RPA-T8), anti-CD16 (55661), anti-CD45RA (HI1100), and anti-CD19 (H1B19); PE-conjugated anti-CD14 (55715) and anti-CD4 (OKT4); and APC-conjugated anti-CD3 (UCHT1), anti-HLA-DR (LN3), and anti-CD45R0 (UCHL1).

Stained mouse and human leukocytes were evaluated by FCM analysis using a FACSCalibur flow cytometer interfaced with CellQuest software (BD-Biosciences, Immunocytometry Systems). Electronic bit maps were utilized to encompass and gate lymphocyte and monocyte subsets during FCM analysis.

### Measures of Lymphocyte Apoptosis and Necrosis

Spleen cells from 14 weeks old Wt and Tg mice were evaluated as fresh isolates or were stimulated for 24 or 48 hrs as for lymphocyte proliferation. Harvested spleen cells were stained with annexin-V-FITC (Apoptosis Detection kit, Calbiochem/EMD Biosciences, Inc., San Diego, CA), PE-anti-Thy-1 (clone 53-2.1) to detect T cells, and APC anti-CD45R/B220 (clone RA3-6B2) to detect B cells (eBioscience). Actinomycin D (7-ADD; BD Pharmingen) was used as a viable exclusion indicator for membrane permeability to distinguish apoptotic (annexin-V^+^7-ADD^−^) from necrotic cells (annexin-V^+^7-ADD^+^), the latter having lost membrane integrity.

### Isolation and Purification of CD4^+^CD25^+^ (Treg) and CD4^+^CD25^−^ (Teff) Cells

Treg and Teff cells were isolated as previously described [44]. Lymph nodes (cervical, mandibular, axillary, brachial, inguinal and mesenteric) and spleens were harvested from male Wt B6 mice (9 weeks old). After lysis of red blood cells, T cell populations were enriched by negative selection on CD3^+^ T cell columns (R&D Systems, Minneapolis, MN). CD3^+^ T cells were further passed through CD4^+^ T cell subset enrichment column (R&D Systems) to obtain a highly pure CD4^+^ T cell population in the eluted fraction. The CD4^+^ T cell fraction was incubated with PE-labeled anti-CD25 antibody (BD Pharmingen) followed by anti-PE microbeads (Miltenyi Biotec, Auburn, CA) and subjected to magnetic separation (Auto MACS, Miltenyi Biotec). Nonadherent cells were eluted from the magnetic column and were enriched for CD4^+^CD25^−^ Teff cells, while adherent cells eluted from the column were enriched as CD4^+^CD25^+^ Treg cells. Purity of nonadherent and adherent cell fractions were determined by FCM analysis (FACSCalibur flow cytometer, BD Biosciences) using antibodies that recognize disparate epitopes to CD3, CD19, CD4, CD8, CD25, and Foxp3 (eBioscience). Prior to activation, fresh isolates of Tregs were >95% CD4^+^CD25^+^Foxp3^+^ while Teff were >95% CD4^+^CD25^−^Foxp3^−^. To activate and expand enriched T cell populations, purified cells were cultured for 4 days in 24-well plates at 1×10^6^ cells per ml of complete RPMI 1640 with 0.5 µg/ml anti-CD3 (145-2C11; BD Pharmingen) and 3×10^6^ irradiated splenocytes (3,300 rads). CD4^+^CD25^+^ T cells required the addition of 100 U/ml of mouse recombinant interleukin (IL)-2 (R&D Systems) [44]. Furthermore, CD4^+^CD25^+^ Tregs exhibited increased expression of mRNA for Foxp3, TGF-β and IL-10, whereas Teff showed increased expression of IFN-γ mRNA. Tregs also inhibited anti-CD3 induced mitogenesis in a dose-dependent manner (data not shown).

### Adoptive Cell Transfers

Freshly isolated lymphocytes obtained from spleens of naïve Wt B6 donor mice and anti-CD3 activated Treg or Teff cells after 4 days of stimulation *in vitro* were harvested, washed, and resuspended in HBSS. To B6 SOD1 Tg recipient mice, 50×10^6^ lymphocytes or 1×10^6^ Treg or Teff cells in 0.25 ml of HBSS or PBS alone were administered intravenous every 6 weeks at 7, 13, and 19 weeks of age.

### Body Weight and Clinical Signs

The initial sign of the disease is a high frequency resting tremor that progresses to gait impairment, asymmetrical or symmetrical paralysis of the hind limbs, followed by complete paralysis at end stage. Beginning at 7 weeks of age, all animals were assessed weekly for body weight and for signs of motor deficit with the following 4 point-scoring system: 4 points if normal (no sign of motor dysfunction), 3 points if hind limb tremors were evident when suspended by the tail, 2 points if gait abnormalities were present, 1 point for dragging of at least one hind limb, and 0 point for symmetrical paralysis [modified from [45]. Disease onset was determined at the earliest presentation of symptoms (i.e. score = 3). Mice that reached a clinical score of 0 or lost 20% of maximum body weight were deemed unable to survive, removed from the study, immediately euthanized, and scored as a terminal event.

### Paw Grip Endurance (PaGE) Test

Grip strength of hind limbs of mice were assessed each week as previously described [45]. Each mouse was placed on the wire-lid of a conventional housing cage and gently shaken to prompt the mouse to hold on to the grid. The lid was turned upside down and the duration determined until the mouse released both hind limbs. Each mouse was given three attempts with a maximum duration of 90 sec and the longest latency was recorded.

### Rotarod Performance

Mice were pre-conditioned for 3 days prior to testing then monitored for rotarod performance once every week starting at 7 weeks of age [39]. In brief, mice were placed on a partitioned rotating rod (Rotamex Rota-rod apparatus, Columbus Instruments, Columbus, OH) and tested at a 5, 10, and 15 rpm for a maximum of 90 sec at each speed with a minimum of 5 min rest between attempts. The overall rotarod performance (ORP) was calculated as the area under the curve using Prism (version 4, Graphpad Software, San Diego, CA) from the plot of the time that the animal remained on the rod as a function of the rotation speed

### Statistical Analyses

All values are expressed as mean±SEM. Differences among normally distributed means were evaluated by Student's *t* test for two group comparisons or one-way ANOVA followed by Bonferroni or Fisher's LSD *post-hoc* tests for pairwise comparisons amongst multiple data sets exhibiting equal variances or by Dunnett's *post-hoc* tests for data exhibiting unequal variances (Statistica v7, StatSoft, Tulsa, OK, and SPSS v13, SPSS, Inc., Chicago, IL). Cox's F-test comparison was performed for comparison between treatment groups for Kaplan-Meier analyses.

## Results

### COP-1 Immunization of B6 SOD1 Tg Mice

**Figure 1:**
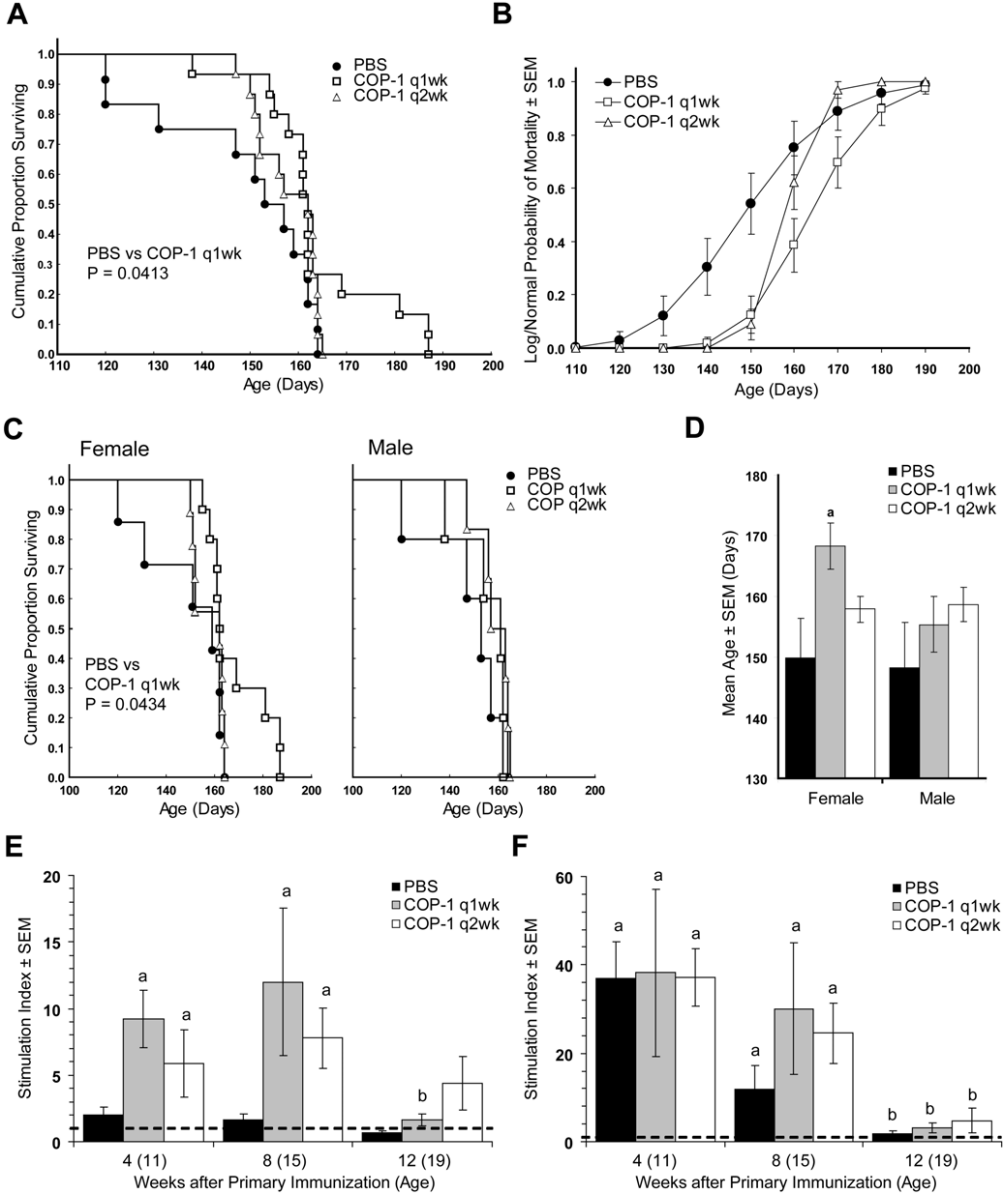
Effect of COP-1 immunization in B6 SOD1 mice. Mice were treated with PBS (closed circles and black bars), COP-1 weekly (q1wk) (open boxes and gray bars), or COP-1 every 2 weeks (q2wk) (open triangles and white bars). (A) Kaplan-Meier analysis of the proportion of surviving SOD1 Tg mice as a function of age. Cox's F-test comparison showed groups treated with PBS vs COP-1 q1wk (p = 0.0413) or COP-1 q2wk (p = 0.1151), and COP-1 q1wk vs COP-1 q2wk (p = 0.1673). (B) Log-normal analysis of mortality probability at 10 day intervals for mice treated with PBS, COP-1 q1wk, COP-1 q2wk. (C) Kaplan-Meier plot of the proportion of surviving female SOD1 Tg mice (left panel) or male SOD1 Tg mice (right panel) as a function of age showing the gender effect. Cox's F-test comparison showed female mice groups treated with PBS vs COP-1 q1wk (p = 0.0434) or COP-1 q2wk (p = 0.2449), and COP-1 q1wk vs COP-1 q2wk (p = 0.0846) and male mice groups treated with PBS vs COP-1 q1wk (p = 0.4240) or COP-1 q2wk (p = 0.1615), and COP-1 q1wk vs COP-1 q2wk (p = 0.1430). (D) Mean age of survival±SEM for 7–10 female or 5–6 male SOD1 Tg mice/group treated with PBS, COP-1 q1wk, or COP-1 q2wk. ^a^P<0.05 compared to PBS treated mice with Bonferroni *post-hoc* tests. Spleen cells from B6 Tg mice treated with PBS, COP-1 q1wk, or COP-1 q2wk were stimulated with (E) Cop-1 (5 µg/ml), (F) Con A (2 µg/ml) or cultured in media alone. Cells were pulsed with [^3^H]-TdR for the final 18 hrs of culture, harvested onto glass fiber filters and counted by β-scintillation spectrometry. Counts were normalized as a ratio of those obtained from culture in media alone to generate a stimulation index for spleen cell proliferation from each animal. A stimulation index of 1 is defined by spleen cells cultured in media alone (dashed line). Means of stimulation indices (±SEM) were determined from 4–5 mice/group for (E) antigen-specific proliferation elicited by Cop-1 and (F) polyclonal T cell proliferation induced by the T cell mitogen, Con A. ^a^P<0.05, above media control (stimulation index = 1, dashed line); and ^b^P<0.05 compared to weeks 4 or 8 within each treatment group.

Our initial works investigated whether COP-1 immunization of B6 SOD1 Tg mice affect disease progression. In these experiments we immunized male and female B6 SOD1 Tg mice with 75 µg COP-1 s.c. in 0.1 ml PBS either every week (q1wk) or every other week (q2wk), or animals were treated every week with PBS as excipient controls. Kaplan-Meier analysis indicated that weekly COP-1 immunization had an affect on the lifespan of SOD1 Tg mice compared to PBS controls (p = 0.0413), however immunization every other week did not increase survival (p = 0.1673) (Fig. 1A). For mice immunized weekly with COP-1, the mean age of survival increased by 9.9% compared to PBS controls (p = 0.006), while COP-1 immunization every other week increased the mean age of survival by 6.1%, however this did not reach significance (data not shown). Log-normal analysis of mortality probability at 10 day intervals showed that COP-1 immunization every week and every other week initially provided protective benefits compared to PBS controls; however, by 160 days of age, the probability of mortality for mice immunized every other week evolved to that afforded by PBS controls (Fig. 1B). Kaplan-Meier analysis of treated mice stratified for gender indicated that increased survival by weekly immunization was associated with female mice (p = 0.0434), but had no effect on survival of male Tg mice (Fig. 1C). Similarly, compared to PBS treated controls, immunization with COP-1 every week increased the mean age of survival for female, but not male Tg mice, and immunization every other week produced no difference in mean age of survival for either male or female Tg mice (Fig. 1D). These results posed the question as to whether adaptive immunity was fully functional in pre-symptomatic SOD1 Tg mice.

### Impaired T Cell Immune Responses in SOD1 Tg Mice

Based on the failure of the COP-1 immunization strategies to increase longevity in male SOD1 Tg mice, and preliminary data showing diminished spleen size and immune responses with age, we tested whether T cell responses were functional. These studies revealed that T cell immune function elicited in B6 SOD1 Tg male mice was significantly impaired by 19 weeks of age. Spleen cells from 4 and 8 week-immune SOD1 Tg mice, stimulated *in vitro* with COP-1 exhibited increased stimulation indices compared to those cultured in media alone (dashed line), whereas cells from PBS treated mice were unable to respond to COP-1 (Fig. 1E) indicating that immunization strategies elicited functional COP-1 responsive T cells in early stage of the disease. However, after 12 weeks of weekly or bi-weekly immunizations, stimulation indices of COP-1 stimulated spleen cells diminished to levels statistically indiscernible from those of cells cultured in media alone indicating that the T cell immune responses in those mice had waned. In concomitant assays to test the overall functionality of all T cell populations, we stimulated spleen cell cultures with Con A, a T cell mitogen. Stimulation indices of Con A induced T cells from B6 SOD1 Tg mice in all treatment groups after 4 and 8 weeks (at 11 and 15 weeks of age) were significantly above those of media control cells (dashed line) (Fig. 1F), demonstrating the presence of functional T cells in those mice. However in Tg mice at 19 weeks of age, after 12 weeks of treatment, stimulation indices of Con A stimulated T cells were indistinguishable from those cultured in media alone. Regression analysis of stimulation indices of Con A stimulated T cells from PBS controls indicated a progressively diminished proliferative capacity of T cells that was strongly associated with increasing age of B6 SOD1 Tg mice (r^2^ = 0.6308, p = 0.002). Taken together these results suggest a global dysregulation of T cell function with age in SOD1 Tg mice.

### Spleen Size, Weight and Cell Counts in SOD1 Tg Mice

**Figure 2:**
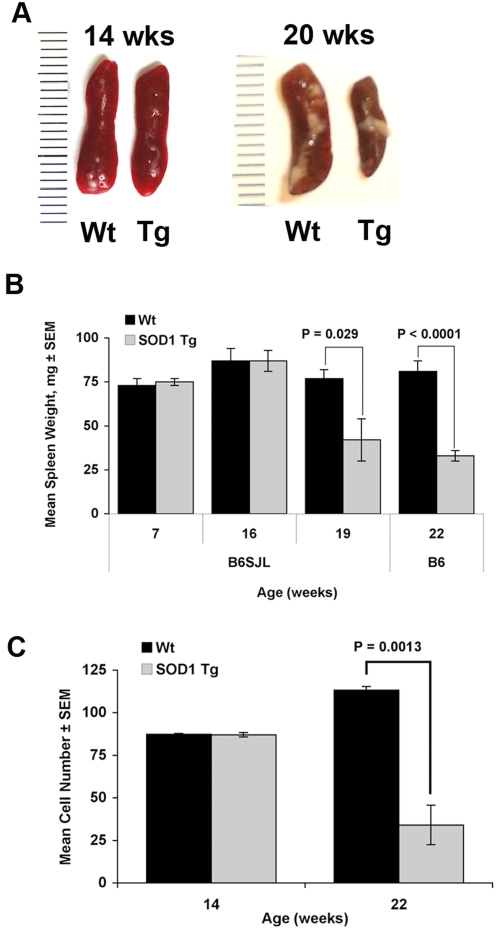
Spleen changes in G93A-SOD1 Tg mice. (A) Morphology and size of spleens from B6SJL SOD1 Tg mice and Wt littermates at 14 weeks of age (left panel) and 20 weeks of age (right panel). (B) Mean spleen weights were compared between B6SJL Wt littermates and B6SJL SOD1 Tg mice at 7, 16 and 19 weeks age and between B6 Wt and B6 SOD1 Tg mice at 22 weeks age (n = 5–9 mice/group). (C) Total spleen cell numbers were compared between Wt littermates and B6SJL SOD1 Tg mice at 14 and 22 weeks of age. Values are means±SEM for 3–9 mice per group.

Based on marginal protection achieved by COP-1 immunization and progressively diminished T cell function with age, we next investigated the adaptive immune system in disease whereby spleens from B6SJL G93A-SOD1 Tg mice were compared at early symptomatic stage (14 weeks of age) and end stage (20–22 weeks of age) with those of age and sex-matched Wt littermate controls. All Tg mice at 14 weeks of age exhibited hind limb tremors. Morphologically, spleens from 14 weeks old B6SJL Tg mice were identical to those of Wt controls (Fig. 2A, left panel), whereas, spleens from end stage mice showed marked reduction in size compared to controls (Fig. 2A, right panel). Similarly, no differences in spleen weights from pre-symptomatic and symptomatic B6SJL mice compared to Wt were discerned, whereas at end stage, spleen weights were diminished by 45% in B6SJL Tg mice (19 weeks old) and by 59% in B6 Tg mice (22 weeks old) (Fig. 2B). No differences in gross morphology or weights for non-lymphoid kidneys or livers were discernible between Tg and Wt mice at any age (data not shown). For end stage B6 Tg mice, total viable spleen cell numbers were diminished by 70% compared to Wt controls, whereas no differences in spleen cell numbers were observed between Tg and Wt mice in early symptomatic stage (Fig. 2C).

### Immune Tissue Analyses of SOD1 Tg Mice

**Figure 3:**
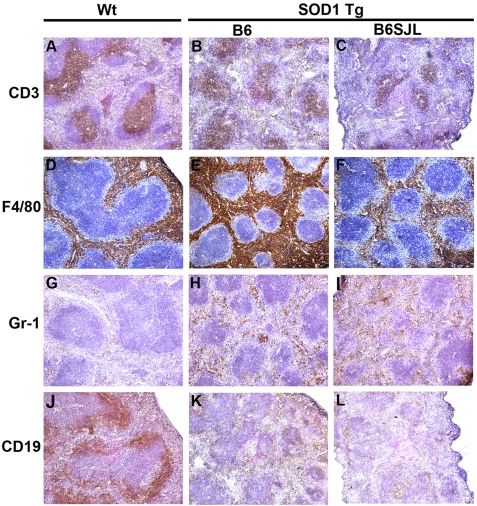
Altered spleen architecture from end stage G93A-SOD1 Tg mice. Representative photomicrographs of immunohistochemistry are shown for expression of CD3, F4/80, Gr-1, CD19 of fresh frozen spleen sections from end stage SOD1 Tg mice and age-matched Wt controls. Photomicrographs in the left panels are from B6 Wt mice, while middle and right panels show sections from B6 SOD1 Tg and B6SJL SOD1 Tg mice, respectively. Sections are stained by immunoperoxidase (brown) for expression of (A, B, C) CD3 by T cells; (D, E, F) F4/80 by perifollicular macrophages; (G, H, I) Gr-1 immunoreactivity on myeloid cells; and (J, K, L) CD19^+^ on B cells. Sections are counterstained with hematoxylin (blue).

To assess splenic architecture in end stage mice, we assessed expression of CD3, CD19, F4/80, and Gr-1 in fresh frozen sections from end stage B6 Tg mice (22 weeks old), B6SJL Tg mice (19 weeks old), and age-matched Wt controls. Splenic architecture in end stage B6 Tg and B6SJL Tg mice revealed remarkable alterations in follicle number, size, and expression of hematopoietic lineage markers compared to Wt B6 littermates. Splenic follicular architecture appeared diminished with a greater number of follicles in each field for B6 SOD1 Tg (Fig. 3B, 3E, 3H, and 3K) and B6SJL SOD1 Tg (Fig. 3C, 3F, 3I, and 3L) mice compared to Wt controls (Fig. 3A, 3D, 3G, and 3J). The density of CD3^+^ T cells in the spleen appeared unaltered in B6 Tg (Fig. 3B) or B6SJL Tg (Fig. 3C) mice compared to Wt mice (Fig. 3A), while expression of F/480 (Fig. 3D, 3E, and 3F) and Gr-1 (Fig. 3G, 3H, and 3I) in the perifollicular area of spleen appeared increased in SOD1 Tg mice and the density of CD19 expression by B cells within the follicles of SOD1 Tg mice (Fig. 3K and 3L) appeared diminished compared to Wt controls (Fig. 3J) .

**Figure 4:**
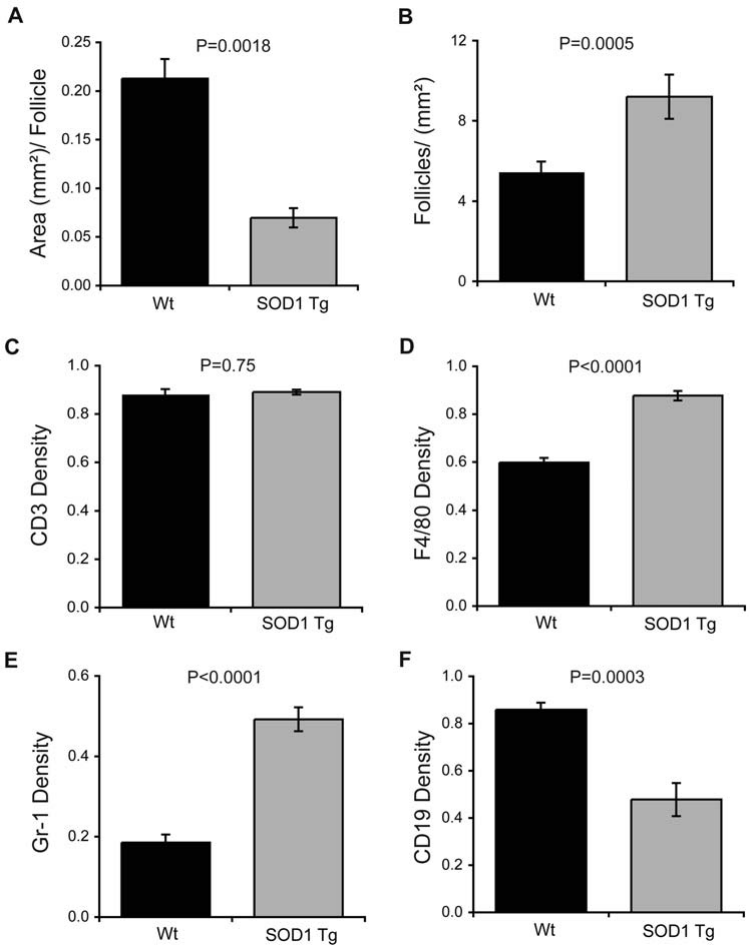
Comparison of spleen architecture between Wt and SOD1 B6 Tg mice (22 weeks old). (A) Mean area/follicle and (B) mean numbers of follicles/mm^2^ were determined for B6 Wt and B6 SOD1 Tg mice from digital images taken at 100× magnification (4 random fields/mouse). Densities of (C) CD3^+^ T cells, (D) F4/80^+^ macrophages, (E) Gr-1^+^ cells, and (F) CD19^+^ B cells from concomitantly stained sections were determined by digital image analysis from 100× magnifications using Image-Pro Plus software. Values are means±SEM for 3–6 mice per group.

These observations were validated by digital image analysis in B6 Wt and B6 SOD1 Tg mice. In Tg mice, splenic follicular area was diminished by 67% (Fig. 4A) and numbers of follicles/mm^2^ were increased by 41% (Fig. 4B) compared to Wt controls. No difference in the densities of splenic CD3 expression could be ascertained between Tg and Wt control mice (Fig. 4C). In the splenic perifollicular area of Tg mice compared to Wt controls, the mean density of F4/80 was increased by 47% (Fig. 4D), density of Gr-1 expressing cells was increased by 165% (Fig. 4E), while the intrafollicular density of CD19 expression was diminished by 88% (Fig. 4F).

### Impaired Lymphocyte Proliferation and Necrosis in Spleens of SOD1 Tg Mice

**Figure 5:**
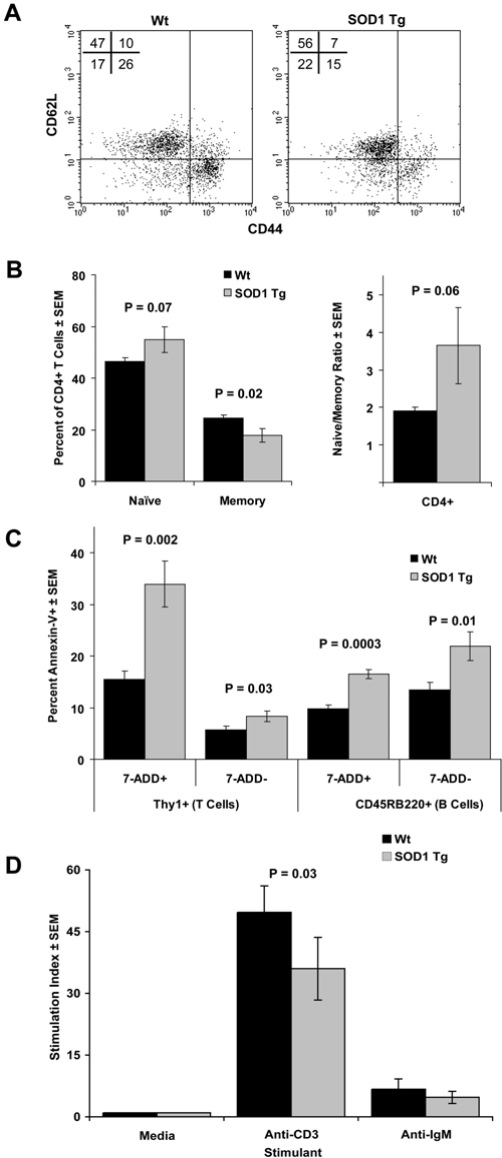
Lymphocyte phenotype and function in G93A-SOD1 Tg mice. The phenotype and function of splenic lymphocytes from B6SJL SOD1 Tg and Wt littermates were assessed by flow cytometric analysis (FCM) and proliferation assays. (A) Representative dot plot for FCM analysis of CD4^+^ gated naïve (CD44^lo^CD62L^hi^) and memory (CD44^hi^CD62L^lo^) T cells from Wt (left) and SOD1 Tg (right) mice at 14 weeks of age. (B) Mean percentages (±SEM) of CD4^+^ naïve and memory T cells (left panel) and ratios of naïve/memory CD4^+^ T cells (right panel) were determined for 5 Wt and 5 B6SJL SOD1 Tg mice. (C) Percentages of annexin-V^+^7ADD^+^ (necrotic) and annexin-V^+^7ADD^−^ (apoptotic) Thy-1^+^ T cells or CD45RB220^+^ B cells amongst splenic lymphocytes were assessed in Wt and B6SJL SOD1 Tg mice at 14 weeks of age. (D) Lymphoproliferative responses of Wt littermates and B6SJL SOD1 Tg mice at 14 weeks of age were evaluated after *in vitro* stimulation for 3 days with anti-CD3 (1 µg/ml), anti-IgM (20 µg/ml), or media alone. Stimulation indices for [^3^H]-TdR uptake by splenocytes from each animal were determined from quadruplicate cultures and values represent the mean±SEM for 5 mice per group.

Based on diminished T cell responses and observations of profound lymphopenia in G93A-SOD1 Tg mice at end stage, we assessed splenic lymphocyte phenotype and function in early symptomatic (14 weeks old) B6SJL SOD1 Tg mice to detect early immune cell aberrations in spleen. Flow cytometric analysis of CD62L and CD44 expression on CD4^+^ gated splenic lymphocytes (Fig. 5A) demonstrated a diminished percentage of CD4^+^CD44^hi^CD62L^lo^ memory T cells and an increased percentage of CD4^+^CD44^lo^CD62L^hi^ naïve T cells compared to Wt mice which resulted in an increased ratio of naïve/memory CD4^+^ T cells in Tg mice compared to Wt controls (Fig. 5B). To assess lymphocytic demise, we evaluated apoptotic (annexin-V^+^7-ADD^−^) and necrotic (annexin-V^+^7-ADD^+^) lymphocytes among viable T cells (Thy-1^+^) and B cells (CD45R/B220^+^) from spleen cell isolates of early symptomatic (14 weeks old) B6SJL SOD1 Tg mice and Wt controls. Flow cytometric analysis revealed that Tg mice had a greater than 2-fold increase in the percentage of annexin-V^+^7-ADD^+^ necrotic splenic T cells and a 30% increase in the percentage of annexin-V^+^7-ADD^−^ apoptotic T cells compared to Wt littermates (Fig. 5C). Similarly, percentages of necrotic (41%) and apoptotic (38%) B cells were increased in Tg mice compared to control mice.

To assess lymphoid cell function of early symptomatic B6SJL SOD1 Tg mice at 14 weeks of age, we stimulated T cells with anti-CD3 and B cells with anti-IgM and evaluated their proliferative capacity of each lineage. T cell proliferation in B6SJL Tg mice was significantly diminished compared to Wt littermates; however no diminution of B cell function could be ascertained (Fig. 5D). The diminished T cell proliferative responses thus confirmed our previous findings (Fig. 1F). We also assessed whether lymphoid cells from Tg and Wt mice were differentially susceptible to activation-induced cell death at either 24 or 48 hrs post-activation, however no differences in induction of apoptotic or necrotic T or B cells at any time point after activation were observed (data not shown).

### Survival of B6 SOD1 Tg Mice after Adoptive Transfer of Naïve Lymphoid Cells, or Activated Treg or Teff Subsets

**Figure 6:**
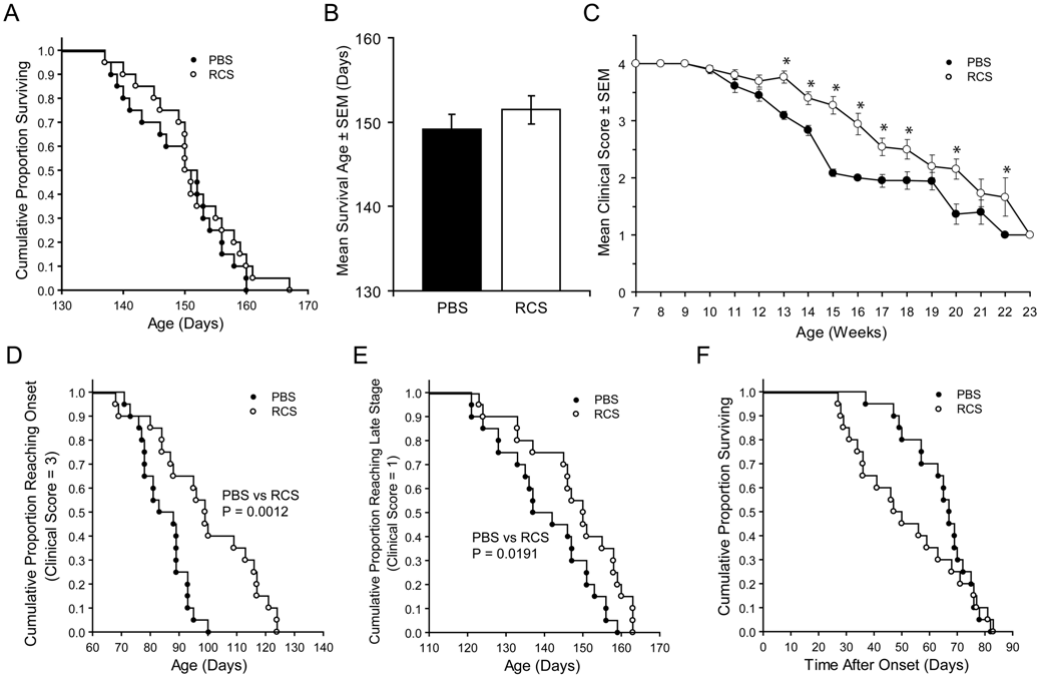
Effect of total lymphocyte reconstitution on survival and clinical scores in B6 SOD1 Tg mice. B6 SOD1 mice (20 mice/group) were treated with PBS (closed circles) or RCS (open circles) with 50×10^6^ naïve splenic lymphocytes. (A) Kaplan-Meier analysis of the proportion of surviving SOD1 Tg mice as a function of age. P = 0.2035 by Cox's F-test for comparison of PBS and RCS groups. (B) Mean age of survival±SEM for 20 mice/group treated with PBS (black bar, 149.3±7.5 days) or reconstituted with naïve lymphocytes (RCS) (white bar, 151.5±7.5 days). Comparison of treatment groups indicated p = 0.315 by ANOVA. (C) Mean clinical scores (±SEM) of PBS- or RCS-treated SOD1 Tg mice as a function of age in weeks. ^*^P<0.05 compared to PBS treated group by factorial ANOVA and Fisher's LSD *post-hoc* tests of treatment and age. (D) Kaplan-Meier analysis of age and cumulative proportion of SOD1 Tg mice reaching onset of disease (clinical score = 3). P = 0.0012 by Cox's F-test comparison of reconstituted mice to those treated with PBS. (E) Kaplan-Meier analysis of age and proportion of SOD1 Tg reaching late disease stage (clinical score = 1). P = 0.0191 by Cox's F-test comparison of reconstituted mice to those treated with PBS. (F) Kaplan-Meier analysis of the cumulative proportion of SOD1 Tg mice surviving after the time of disease onset (clinical score = 3). P = 0.2021 by Cox's F test comparison of RCS and those mice treated with PBS.

Based on the above findings in SOD1 Tg mice demonstrating a lack of protective response by COP-1 immunization in male mice, diminished T cell functional capacity in early symptomatic and late stage mice, and end stage lymphopenia, we tested a strategy to rectify the lymphoid dysregulation and extend survival by adoptive transfer of B6 Wt naïve lymphoid cells to recipient B6 SOD1 Tg mice. B6 SOD1 Tg mice treated with 50×10^6^ naive spleen cells at 7, 13, and 19 weeks of age yielded no significant differences in the cumulative proportion of survival (Fig. 6A, p = 0.2035) or mean age of survival (Fig. 6B, p = 0.315) compared to PBS-treated mice. However, mean clinical scores analyzed by factorial ANOVA revealed significant improvement of reconstituted mice compared to PBS-treated controls (Fig. 6C, p = 0.000001). Additionally, Kaplan-Meier analysis showed immune reconstituted (RCS) Tg mice exhibited delayed symptom onset (clinical score = 3) (Fig. 6D, p = 0.0012) as well as delayed entry into late stage (clinical score = 1) (Fig. 6E, p = 0.0191). However, after onset of symptoms, survival of reconstituted Tg mice trended to be diminished compared to the PBS controls as determined by Kaplan-Meier analysis (Fig. 6F, p = 0.2021) and by mean latency after onset to death for PBS-treated (64.5±2.6) and RCS (52.0±4.3) mice (p = 0.0167) (data not shown).

No significant differences in body weight were discerned between RCS- and PBS-treated groups as a function of age by factorial ANOVA (Fig. S1A, p = 0.5824). Additionally, no differences in PBS-treated or RCS groups were found in the cumulative proportion (p = 0.2744) and the mean age (p = 0.2921) of Tg mice that reach 10% loss of maximum body weight (data not shown). Although an early effect in hind grip strength was observed between 10–13 weeks of age as determined by PaGE, no effects were discernible thereafter (Fig. S1B). Factorial ANOVA indicated no differences in motor function as determined by ORP in PBS-treated or RCS groups over their lifetime (Fig. S1C, p = 0.8862).

**Figure 7:**
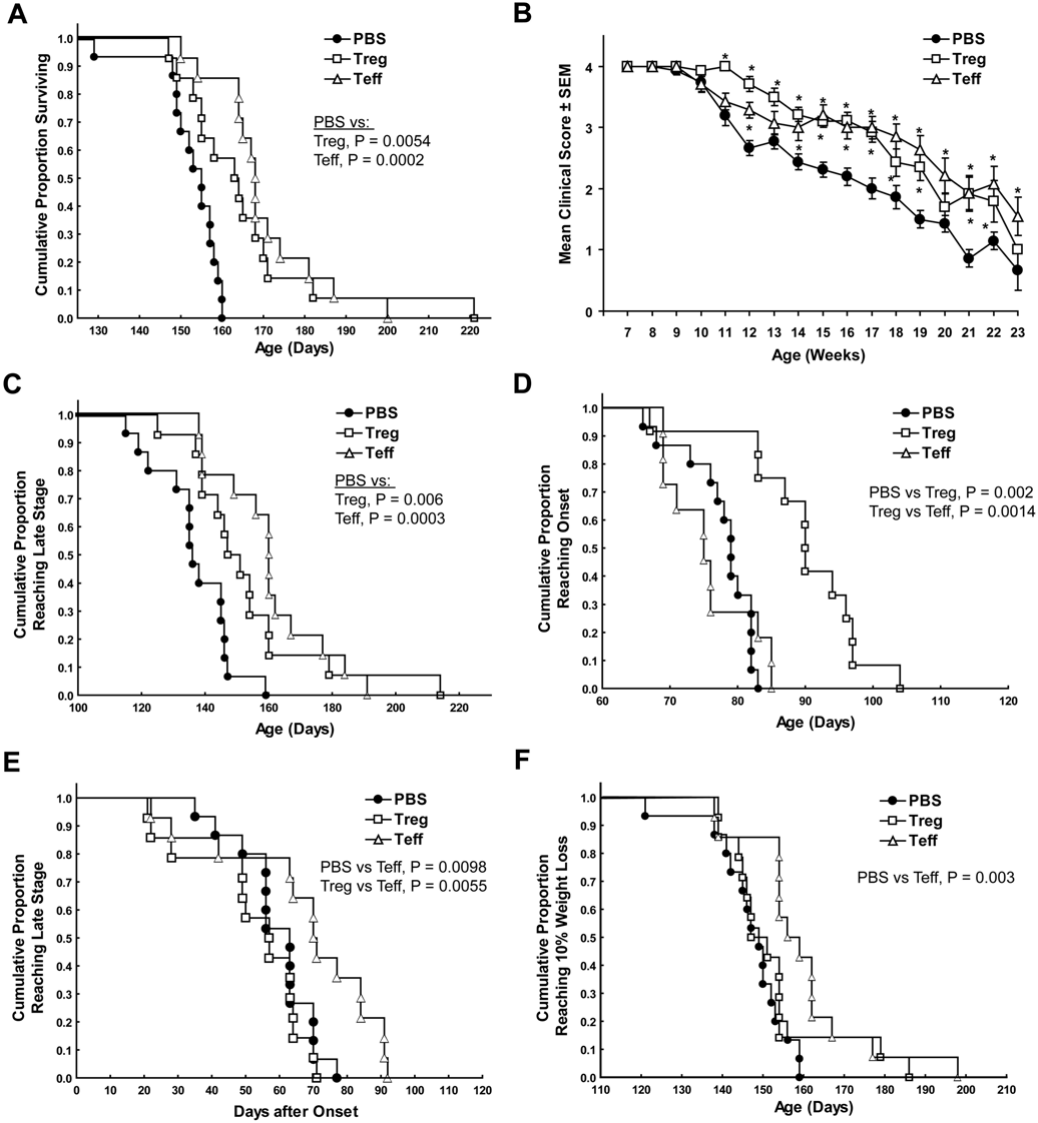
Effect of Treg and Teff on survival, clinical scores and weight loss in B6 SOD1 Tg mice. B6 G93A-SOD1 mice (14–15 mice/group) were treated with PBS (closed circles), 1×10^6^ activated Treg (open boxes), or 1×10^6^ activated Teff (open triangles) at 7, 13, and 19 weeks of age. (A) Kaplan-Meier analysis of the proportion of surviving SOD1 Tg mice as a function of age. Cox's F-test comparison of groups treated with PBS vs Treg (p = 0.0054) or Teff (p = 0.0002), and Treg vs Teff (p = 0.2505). (B) Clinical scores of SOD1 Tg mice as a function of age in weeks. *P<0.05 compared to PBS treated group at each time point by factorial ANOVA and Fisher's LSD *post-hoc* tests. (C) Kaplan-Meier analysis for age and proportion of SOD1 Tg reaching late disease stage (clinical score = 1). Cox's F-test comparison of groups treated with PBS vs Treg (p = 0.006) or Teff (p = 0.0003), and Treg vs Teff (p = 0.1883). (D) Kaplan-Meier analysis of age and cumulative proportion of SOD1 Tg mice reaching onset of disease (clinical score = 3). Cox's F-test comparison of groups treated with PBS vs Treg (p = 0.002) or Teff (p = 0.4215), and Treg vs Teff (p = 0.0014). (E) Kaplan-Meier analysis of the cumulative proportion of SOD1 Tg mice and the time after disease onset (clinical score = 3) to reach late stage (clinical score = 1). Cox's F test comparison of groups treated with PBS vs Treg (p = 0.2716) or Teff (p = 0.0098), and Treg vs Teff (p = 0.0055). (F) Kaplan-Meier analysis of the age and the cumulative proportion of SOD1 Tg mice that exhibited a reduction of maximum body weight ≥10%. Cox's F-test comparison of groups treated with PBS vs Treg (p = 0.2131) or Teff (p = 0.003), and Treg vs Teff (p = 0.0807).

**Figure 8:**
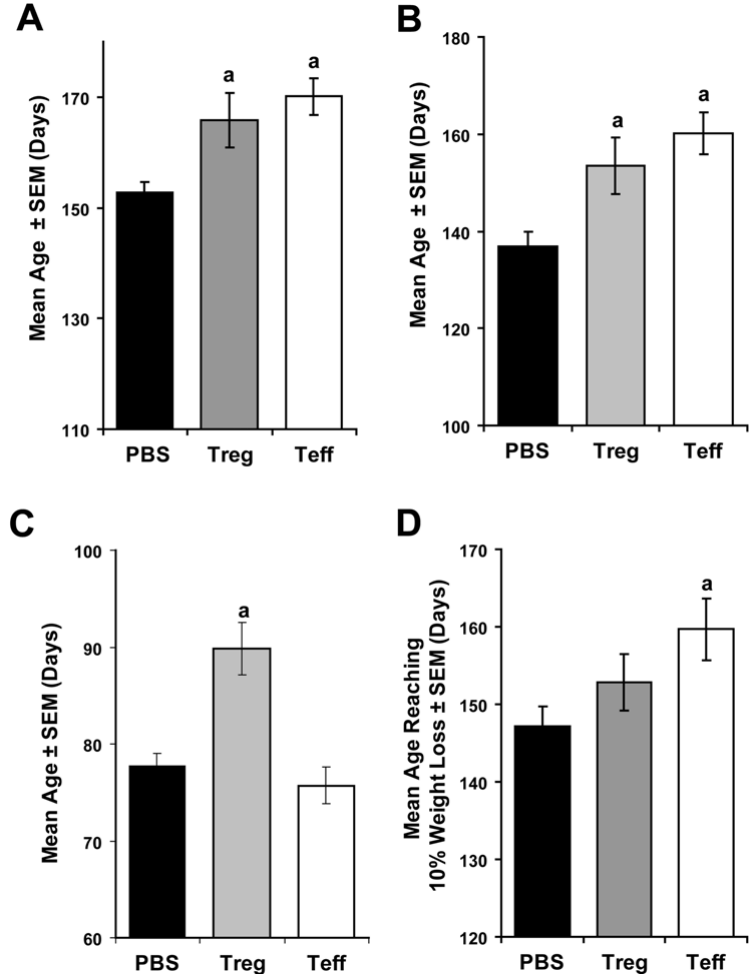
Effect of Treg and Teff on mean age of survival, clinical scores and weight loss in B6 G93A-SOD1 Tg mice. B6 G93A-SOD1 mice were treated with PBS (black bars), 1×10^6^ activated Treg (gray bars), or 1×10^6^ activated Teff (white bars) at 7, 13, and 19 weeks of age. (A) Mean age of survival±SEM for 14–15 SOD1 Tg mice/group treated with PBS (152.7±2.0 days), Treg (165.8±5.0 days), or Teff (170.1±3.4 days). ^a^P<0.04 compared to PBS-treated mice by ANOVA and Bonferroni *post-hoc* tests. (B) Mean age±SEM for SOD1 Tg mice reaching late stage (clinical score = 1) after treatment with PBS (136.9±3.1 days), Treg (153.5±5.8 days), or Teff (160.1±4.3 days). ^a^P<0.04 compared to PBS control group by ANOVA and Bonferroni *post-hoc* tests. (C) Mean age±SEM for SOD1 Tg mice at disease onset (clinical score = 3) after treatment with PBS (77.7±1.3 days), Treg (89.8±2.7 days), or Teff (75.7±1.9 days). ^a^P = 0.0003 compared to PBS control group by ANOVA and Bonferroni *post-hoc* tests. (D) Mean age±SEM for SOD1 Tg mice that exhibit a reduction of maximum body weight ≥10% after treatment with PBS (147.2±2.5 days), Treg (152.8±3.7 days), or Teff (159.7±4.0 days). ^a^P<0.04 compared to PBS control group by ANOVA and Bonferroni *post-hoc* tests.

Based on our previous results which demonstrated that regulatory T cells were neuroprotective in a mouse model of Parkinson's disease [23], [28], [44], we evaluated whether activated T cell subsets also provide analogous protection in SOD1 Tg mice. Adoptive transfer of 1×10^6^ enriched polyclonal-activated Wt Treg (CD4^+^CD25^+^) or Teff (CD4^+^CD25^−^) to B6 SOD1 Tg mice at 7, 13, and 19 weeks of age led to significant increases in longevity as determined by Kaplan-Meier analysis (Fig. 7A) and mean age of survival (Fig. 8A) compared to PBS-treated controls. Factorial ANOVA of treatment and age showed that by 11–12 weeks of age, clinical scores were increased by reconstitution of B6 Tg recipients with activated Wt Treg or Teff compared to PBS controls (Fig. 7B, p = 0.00004). Moreover, entry into late stage disease (clinical score = 1) was delayed by reconstitution with Treg or Teff as determined by Kaplan-Meier analysis (Fig. 7C) and mean age of entry into late stage (Fig. 8B). Of interest, transfer of activated Wt Treg, but not Teff to B6 Tg recipient mice delayed disease onset as evaluated by clinical signs (clinical score = 3) and as determined by Kaplan-Meier analysis (Fig. 7D, p = 0.002) and mean age of disease onset (Fig. 8C, p<0.0003). On the other hand, transfer of activated Wt Teff, but not Treg to B6 Tg recipients, increased the latency from onset (clinical score = 3) to entry into late stage (clinical score = 1) as determined by Kaplan-Meier analysis (Fig. 7E, p = 0.0098). Adoptive transfer of Treg or Teff affected weight gain and loss as determined by factorial ANOVA for effects of treatment as a function of age (Fig. S2A, p = 0.0356). Transfer of activated Teff, but not Treg delayed the age at which recipients lost ≥10% of maximum body weight compared to PBS treatment as determined by Kaplan-Meier analysis of proportion (Fig. 7F, p = 0.003) and mean age (Fig. 8D) of mice reaching ≥10% weight loss.

**Figure 9:**
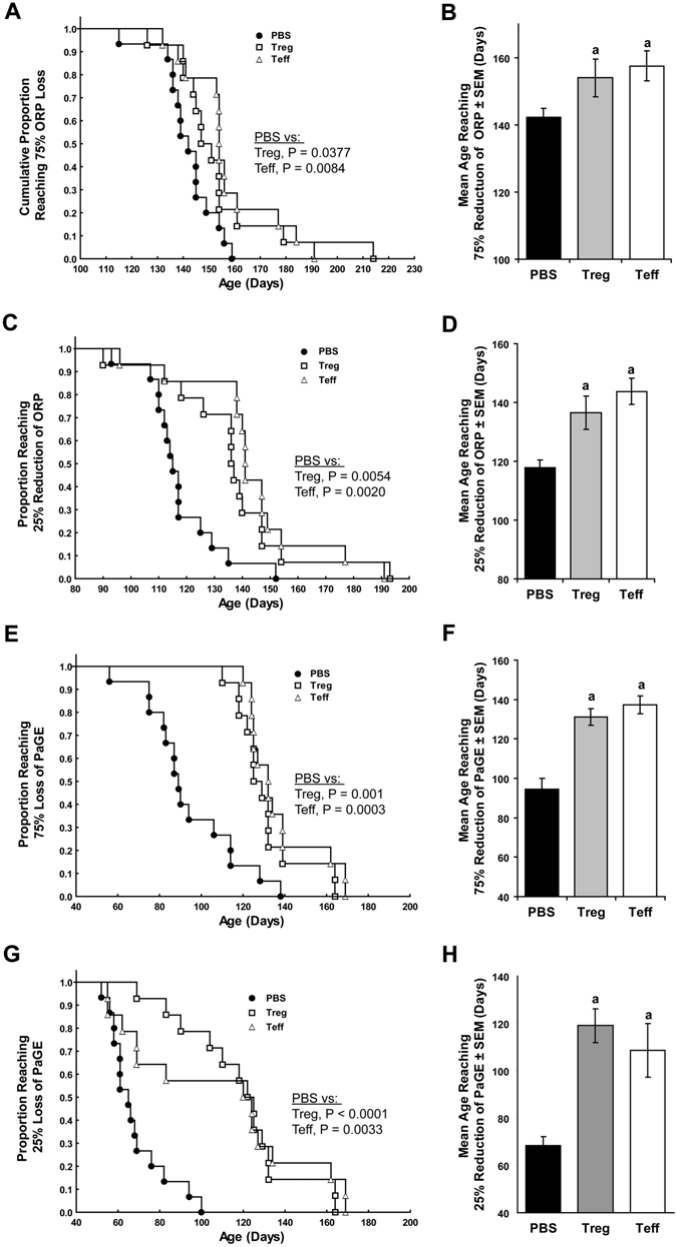
Effect of Treg and Teff on motor function in B6 G93A-SOD1 Tg mice. B6 G93A-SOD1 mice (14–15 mice/group) were treated at 7, 13, and 19 weeks of age with PBS (closed circles and black bars), 1×10^6^ activated Treg (open boxes and gray bars), or 1×10^6^ activated Teff (open triangles and white bars). (A) Kaplan-Meier analysis of the age and the cumulative proportion of SOD1 Tg mice that exhibited a ≥75% reduction of overall rotarod performance (ORP). Cox's F-test comparison of groups treated with PBS vs Treg (p = 0.0377) or Teff (p = 0.0084), and Tregs vs Teffs (p = 0.27). (B) Mean age±SEM for 14–15 SOD1 Tg mice/group at which mice exhibited a ≥75% reduction in ORP after treatment with PBS (142.1±2.8 days), Treg (154.0±5.6 days), or Teff (157.5±4.4 days). ^a^P<0.05 compared to PBS treated mice by ANOVA and Bonferroni *post-hoc* tests. (C) Kaplan-Meier analysis of ages and cumulative proportion of SOD1 Tg mice that exhibited ≥25% reduction in ORP. Cox's F-test comparison of groups treated with PBS vs Treg (p = 0.0054) or Teff (p = 0.0020), and Treg vs Teff (p = 0.20). (D) Mean age±SEM for 14–15 SOD1 Tg mice/group at which mice exhibited ≥25% reduction in ORP after treatment with PBS (117.7±3.5 days), Treg (136.5±6.2 days), or Teff (143.7±6.1 days). ^a^P<0.0025 compared to PBS treated mice by ANOVA and Bonferroni *post-hoc* tests. (E) Kaplan-Meier analysis for age and proportion of SOD1 Tg mice that exhibited ≥75% reduction of maximum Paw Grip Endurance (PaGE) after treatment with PBS, Treg, or Teff. Cox's F test comparison of groups treated with PBS vs Treg (p = 0.001) or Teff (p = 0.0003), and Treg vs Teff (p = 0.14). (F) Mean age±SEM for SOD1 Tg mice that exhibited ≥75% reduction of maximum PaGE after treatment with PBS (94.5±5.6 days), Treg (131.1±4.2 days), or Teff (137.2±4.5 days). ^a^P<0.0001 compared to PBS control group by ANOVA and Bonferroni *post-hoc* tests. (G) Kaplan-Meier analysis for age (days) and proportion of SOD1 Tg mice that exhibited ≥25% reduction of maximum PaGE after treatment with PBS, Treg, or Teff. Cox's F test comparison of groups treated with PBS vs Treg (p<0.0001) or Teff (p = 0.0033), and Treg vs Teff (p = 0.42). (H) Mean age±SEM at which SOD1 Tg mice exhibited at least 25% reduction of maximum PaGE after treatment with PBS (68.5±3.6 days), Treg (119.1±7.2 days), or Teff (108.7±11.3 days). ^a^P<0.002 compared to PBS control group by ANOVA and Dunnett's *post-hoc* tests.

Adoptive transfer of either Treg or Teff to B6 SOD1 Tg mice also improved motor function compared to PBS treated controls as determined by factorial ANOVA for effect of treatment with age on ORP (Fig. S2B, p = 3.3×10^−8^) and PaGE (Fig. S2C, p = 6.7×10^−11^). Activated Wt Treg or Teff delayed loss of rotarod performance as determined by Kaplan-Meier analysis of the proportion (Fig. 9A) and mean age (Fig. 9B) of mice at which ≥75% of ORP was reduced. Also transfer of activated Treg or Teff delayed the initial loss of ORP compared to PBS controls as determined by the cumulative percentage (Fig. 9C) and the mean age (Fig. 9D) of mice that reach ≥25% loss of ORP. We also assessed hind limb strength by PaGE. Compared to PBS controls, adoptive transfer of activated Treg or Teff delayed the loss of hind limb strength as determined by Kaplan-Meier analysis of the cumulative percentage (Fig. 9E) and increased mean age (Fig. 9F) of SOD1 Tg mice exhibiting ≥75% reduction of PaGE. In addition, transfer of activated Wt Treg or Teff to Tg mice delayed early loss of hind limb grip strength compared to controls as determined by Kaplan-Meier analysis of the cumulative percentage (Fig. 9G) and mean age (Fig. 9H) of mice that exhibit ≥25% reduction of PaGE. Of interest, after one round of Treg and Teff adoptive transfer at 49 days of age, Teff appear less efficient than Treg to attenuate early grip loss in Tg mice, however after a second round (at 91 days of age), the capacities to attenuate loss of grip strength by Teff and Treg were comparable (Fig. 9G).

### Preliminary Studies of Altered Adaptive Immunity in ALS Patients

To assess immune alterations in ALS patients, we characterized peripheral blood mononuclear cells (PBMC) from 10 ALS patients and 10 age-matched caregivers. Peripheral blood counts indicated a small, though insignificant increase in the number of leukocytes in patients compared to caregivers. ALS patients exhibited an increase in the mean percentage of polymorphonuclear neutrophils (PMNs) (8.0±0.07×10^9^/L compared with 6.6±0.06×10^9^/L, p = 0.022), with slightly reduced levels of lymphocytes (20.7%±2.4% compared with 25.9%±1.8, p = 0.054), and no discernible differences in monocyte levels (p = 0.35). Flow cytometric analysis showed no differences in levels of peripheral blood CD19^+^ B cells or CD3^+^ T cells among patients and caregivers. However compared to caregivers, ALS patients exhibited an 11.8% decrease in the percentage of CD4^+^CD8^−^ T cells (p = 0.032) and a 22.9% increase in the frequency of CD4^−^CD8^+^ T cells (p = 0.043) compared to age-matched controls. Additionally, the CD45RA/CD45R0 (naïve/memory) ratio among CD4^+^ T cells of ALS patients (0.6±0.1) was diminished by 45% compared to caregivers (1.1±0.2, p = 0.028), which was due to the diminution in levels of CD45RA^+^ naïve T cell among CD4^+^ cells (37.2%±2.5% compared with 47.6%±5.1% for caregivers, p = 0.0435) and a concomitant increase in levels of CD45R0^+^ memory cells among CD4^+^ T cells (62.1%±2.6% compared with 51.7%±5.2% for caregivers, p = 0.0465).

## Discussion

Recent evidence in SOD1 Tg ALS mice suggest neuroimmune dysregulation of microglia that play a role as key contributors of disease progression and in orchestrating motor neuron death [5], [8]–[12], [31]. Activation of glia cells and inflammatory processes coincide with disease onset and progression of disease in SOD1 Tg mice [5], [6], [46]–[49]. Postmortem examinations of ALS neural tissues reveal associations between innate immune and immune effector changes and exhibit reactive microglia, astrocytes, blood-borne macrophages, mast cells, increased number of dendritic cells, elevated chemoattractant factors, major histocompatibility complex (MHC) class I and II molecules, as well as infiltrating CD4^+^ and CD8^+^ T lymphocytes surrounding degenerating neurons and areas affected in ALS [6], [31], [32], [34]–[36]. Systemic immune aberrations in ALS patients include alterations in macrophage activation profiles [41], [42], elevated levels of complement proteins in sera [50], increased IL-13 producing T cells and circulating neutrophils [51], [52], lymphopenia with reduced number of CD2^+^ and CD8^+^ T cells [53]. Studies of human patients with neurodegenerative disorders including ALS, indicate increased expression of molecules associated with adaptive immunity, such as MHC class I, II and human leukocyte antigens in brains, spinal cords, cerebrospinal fluid and sera [54], [55]. Increased levels of monocyte chemoattractant protein (MCP)-1 and RANTES are detected in cerebrospinal fluid and sera from ALS patients [56]–[59]. Several reports support that peripheral immune activation plays a crucial role in AD [15]–[17] and MS [18], [19]. Based on this evidence of concomitant induction of adaptive immune responses and innate immune activation, the possibility of common immune-activating pathways in ALS and other neurodegenerative disorders justified the hypothesis for adaptive immune dysfunction as a major mechanism in neuroimmune dysregulation in ALS. Indeed, we demonstrated that spleens of SOD1 Tg mice at end stage display significant reductions in size and weight and profound lymphopenia. Further, splenic architecture and expression of lymphocyte antigens were markedly altered in SOD1 Tg mice. Moreover, these immune aberrations were preceded by several weeks prior to early disease with significant T cell functional deficits, decreased lymphoproliferative responses *in vitro*, altered ratios of naïve to memory CD4^+^ T cells, and increased percentage of necrotic and apoptotic T cells. Taken together, our data in conjunction with a solitary report showing lymphopenia in peripheral blood of end stage G93A-SOD1 mice [60] strongly indicates the critical involvement of adaptive immune deficits in mutant SOD1-associated pathology, while early presentation of immune dysfunction prior to clinical symptoms and subsequent demise, strongly argues for the possibility that immune dysfunction and loss of adaptive immune cells reflect mechanisms other than those associated with end stage disease. Moreover, these data suggest a plausible explanation for the results observed in the SOD1 Tg mouse in which several drugs previously shown to be efficacious in survival studies, have failed to demonstrate efficacy in human studies [61].

Induction of Treg that secrete anti-inflammatory cytokines and neurotrophic factors [62]–[64], shifts from proinflammatory Th1 to anti-inflammatory Th2 cells [65], and attenuation of microglial responses [23], [28] have been linked to the immunomodulatory action of COP-1. Neuroprotective immunization strategies using COP-1 and COP-1 derivatives administered with or without complete Freund's adjuvant in low or high copy G93A-SOD1 Tg mice have yielded disparate outcomes [37]–[39]. In the present study, COP-1 immunization elicited protection in terms of enhanced survival to female G93A-SOD1 B6 Tg mice. As induction of Th2 cells is one mechanism by which COP-1 mediates neuroprotection and benefits with this strategy have been shown to be limited to female SOD1 Tg mice as shown here and elsewhere [38], this restricted level of neuroprotection was most probably due, at least in part, to diminished T cell immune function in male SOD1 Tg mice as demonstrated by the loss of antigen-induced responses in COP-1 immunized mice and, more so by the global dysfunction of T cell immune responses to the polyclonal stimulators anti-CD3 and Con A in pre-symptomatic and symptomatic stages of the disease. The outcome of COP-1 immunization in male G93A-SOD1 B6 Tg mice in our present study echoes the failure of TV-5010, a COP-1 derivative to enhance survival conducted in three independent studies in G93A B6SJL SOD1, G93A B6 SOD1 and G37R SOD1 mutant mouse models [39].

We initially attempted to correct lymphoid homeostasis and global T cell dysfunction by reconstituting the peripheral immune compartment. However reconstitution of B6 SOD1 Tg mice with Wt naïve splenocytes at regular intervals only transiently delayed clinical onset and entry to late stage, and yielded no significant effect on survival. This indicated that repopulation of the immune compartment, though capable of transient delay of early and late stage disease, was not sufficient to extend survival in SOD1 Tg mice.

As regulatory T cells are known to suppress immune activation, maintain immune homeostasis, attenuate microglia activation and provide beneficial compensation in neurodegeneration [21], [23], [28], [37], [44], [66], [67], we next employed a reconstitution strategy with polyclonal-activated Wt Treg or Teff beginning at the pre-symptomatic stage of SOD1 Tg mice to maintain immune homeostasis and neurological function, and enhance longevity in SOD1 Tg mice. Not only did activated Treg attenuate motor deficits and enhance survival in Tg animals, but activated Teff were equally as effective for these parameters. Interestingly, Treg delayed the onset of symptoms, while Teff cells increased the latency from symptom onset to entry into the terminal phase and also delayed weight loss. Thus, these differential pathways toward a shared protective outcome of disease progression suggest distinct underlying mechanisms conferred by Treg and Teff in neuroprotection with the effects of Treg acting primarily on the afferent response limb. This is congruent with reported activities of CD4+CD25+ Treg and Teff; i.e., Treg typically act on afferent or induction of immune responses, whereas Teff are efferent effectors. Thus neuroprotective Treg and Teff most probably act via several independent pathways. The implication of macrophages and microglia in disease progression in ALS and SOD1 Tg mice [1], [8], [9], [68], [69] and evidence of Treg-mediated regulation of microglia function [21], [44], [70], suggest that Treg affect the induction phase of those cells. Delayed disease onset in SOD1 Tg mice supports that suggestion. On the other hand, the delay of entry to late stage disease after onset by activated Teff cells, suggests that protective populations are acting to attenuate already activated myeloid populations. These results taken together suggest a possible role for polyclonal-activated regulatory T cells in ALS therapy.

Immune dysregulation affecting both adaptive and innate immune systems have a consistent hallmark in ALS [6]. We report for the first time that compared to caregivers, ALS patients exhibited diminished CD45RA/CD45R0 (naïve/memory) ratios within the CD4^+^ T cell subset. Diminished levels of naïve (CD45RA) T cells and increased levels of memory (CD45R0) CD4^+^ T cells were coincidently responsible for diminution of naïve/memory ratios among ALS patients. Evidence of dysregulation within the peripheral adaptive immune system of ALS patients reveals many adaptive immune deficits. Peripheral blood lymphocytes from ALS patients have been reported to exhibit abnormalities in mitochondrial and calcium metabolism [71], reduced levels of metabotropic glutamate receptor 2 mRNA [72], altered expression of antioxidant proteins and responses to nitric oxide [73], [74], and reduced expression of dopamine transporter [75]. More recent evidence that includes increased levels of circulating monocytes and macrophages [41], IL-13 producing T cells [52], and serum anti-Fas antibody levels [76] suggests a more activated peripheral immune system. Although in our patient cohort acute lymphopenia was not evident, the level of lymphocyte frequency trended lower in ALS patients. Nevertheless, evidence linking immunodeficiency in ALS syndromes include the loss of CD8^+^ T cells in ALS patients [53] and the loss of CD4^+^ T cells in HIV-infected patients that displayed ALS-like syndromes [77], [78]. Moreover, disease onset in Guamanian patients exhibiting ALS-inclusive syndromes has been linked to immunodeficiency and loss of T cell-mediated immunity including lymphopenia, diminished frequency and total number of peripheral T cells, diminished responses to skin-test antigens, and decreased T cell responses to mitogens [79]–[81]. These findings are in agreement with loss of T cell function and lymphopenia in SOD1 Tg mice. In contrast to 14 weeks old SOD1 Tg mice, which showed among CD4^+^ T cells, increased naïve and decreased memory T cells, ALS patients in this study, exhibited a significant loss of naïve CD4+ T cells and gain of memory CD4^+^ T cells. The reason for the discrepancy between humans and mice is not clear at this point, however possible explanations include contrasting differences in the murine model of familial ALS and sporadic human ALS, differences in homeostatic mechanism of peripheral blood lymphocytes and murine splenic T cells, or more likely, represents differences in T cell replenishment in relatively younger mice with a functional thymus compared to relatively aged patients in which thymic function is diminished. Nevertheless, compared to normal age-matched controls, our findings emphasize the peripheral immune dysfunction in T cell phenotypes in ALS patients and in SOD1 Tg mice, both T cell phenotype and function.

Here, we present compelling evidence for adaptive immune deficits in ALS. Vaccination with COP-1 to induce regulatory T cells in G93A-SOD1 ALS mice that are genetically encoded for immune deficits, most probably underlies the failure of immune strategies to induce T cells resulting in lack of neuroprotective immune responses in SOD1 Tg mice. Reconstituting a dysfunctional immune system with activated T cell subsets, but not naïve lymphocytes afforded improved neurological function and extended survival in SOD1 Tg mice. Thus, evidence in SOD1 Tg mice of progressive immunological deficits warrants caution in the interpretation of studies utilizing immunopharmacologic interventions in the SOD1 Tg model and in ALS patients.

## Supporting Information

Figure S1 Adoptive transfer of Wt naïve lymphoid cells on weight and motor function of B6 G93A-SOD1 Tg mice. B6 G93A-SOD1 Tg mice (14–15 mice/group) were treated with PBS (closed circles) or 50×10 6 Wt naïve spleen cells (open circles), i.v. (A) Mean body weights of treated SOD1 Tg mice were normalized to percentage of maximum weight (±SEM) and analyzed as a function of age in weeks. Factorial ANOVA of percent maximum body weights did not discern an effect of treatment (p = 0.1424) or of combined treatment and age (p = 0.5824). (B) Mean percentage of maximum paw grip endurance (PaGE)±SEM of treated SOD1 Tg mice were analyzed as a function of age in weeks. Factorial ANOVA did not discern a significant overall effect of treatment (p = 0.5840), but indicated a combined effect of treatment and age between 10 and 13 weeks of age (p = 0.001). *P<0.05 compared to PBS-treated group at each time point by Fisher's LSD post-hoc tests. (C) Mean percentage of overall rotarod performance (ORP)±SEM of treated SOD1 Tg mice were analyzed as function of age in weeks. Factorial ANOVA did not discern an effect of treatment (p = 0.7551) or combined effect of treatment and age (p = 0.8662).
(1.00 MB DOC)

Figure S2 Adoptive transfer of anti-CD3 activated Wt Treg and Teff on weight and motor function of B6 G93A-SOD1 Tg mice. B6 G93A-SOD1 Tg mice (14–15 mice/group) were treated at 7, 13, and 19 weeks of age with PBS (closed circles), 1×106 activated Treg (open boxes), or 1×106 activated Teff (open triangles). (A) Mean body weights of treated SOD1 Tg mice were normalized to percentage of maximum weight±SEM and analyzed as function of age in weeks. Factorial ANOVA of percent maximum body weights indicated a significant combined effect of treatment and age (p = 0.0356). *P<0.05 compared to PBS-treated group at each time point by Fisher's LSD post-hoc tests. (B) Mean percentage of maximum overall rotarod performance (ORP)±SEM of treated SOD1 Tg mice analyzed as function of age in weeks. Factorial ANOVA indicated a significant combined effect of treatment and age (p = 3.3×10−8). *P<0.05 compared to PBS-treated group at each time point by Fisher's LSD post-hoc tests. (C) Mean percentage of maximum paw grip endurance (PaGE)±SEM of treated SOD1 Tg mice were analyzed as function of age in weeks. Factorial ANOVA indicated a significant combined effect of treatment and age (p = 6.7×10−11). *P<0.05 compared to PBS-treated group at each time point by Fisher's LSD post-hoc tests.
(0.30 MB TIF)
