# Supplementary figures and images for: Adaptive Immune Neuroprotection in G93A-SOD1 Amyotrophic Lateral Sclerosis Mice
Source: PLoS One. 2008 Jul 23;3(7):e2740. doi: 10.1371/journal.pone.0002740 (PMC2481277; doi:10.1371/journal.pone.0002740)

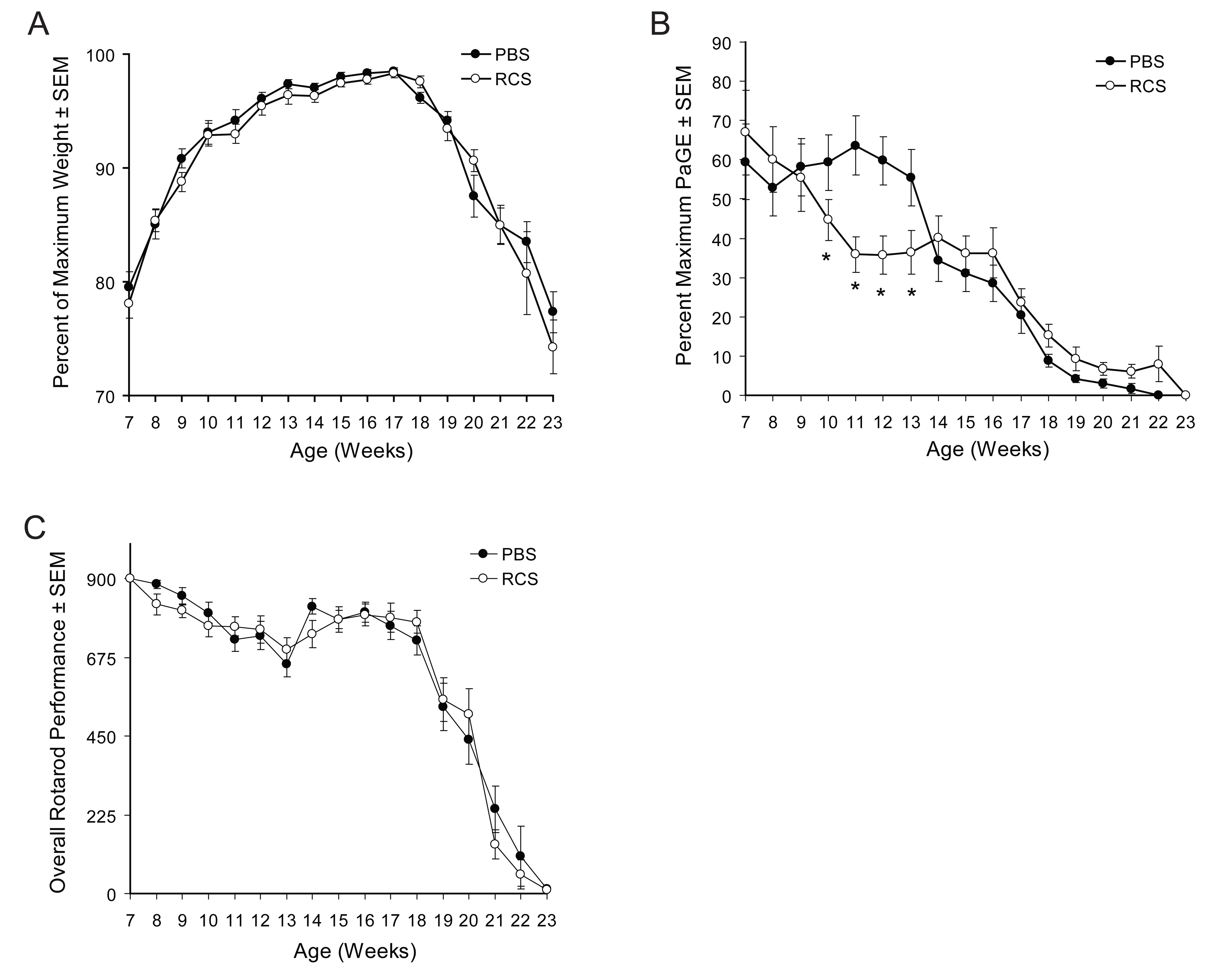

Supplement: Figure S1 — Adoptive transfer of Wt naïve lymphoid cells on weight and motor function of B6 G93A-SOD1 Tg mice. B6 G93A-SOD1 Tg mice (14–15 mice/group) were treated with PBS (closed circles) or 50×10 6 Wt naïve spleen cells (open circles), i.v. (A) Mean body weights of treated SOD1 Tg mice were normalized to percentage of maximum weight (±SEM) and analyzed as a function of age in weeks. Factorial ANOVA of percent maximum body weights did not discern an effect of treatment (p = 0.1424) or of combined treatment and age (p = 0.5824). (B) Mean percentage of maximum paw grip endurance (PaGE)±SEM of treated SOD1 Tg mice were analyzed as a function of age in weeks. Factorial ANOVA did not discern a significant overall effect of treatment (p = 0.5840), but indicated a combined effect of treatment and age between 10 and 13 weeks of age (p = 0.001). *P<0.05 compared to PBS-treated group at each time point by Fisher's LSD post-hoc tests. (C) Mean percentage of overall rotarod performance (ORP)±SEM of treated SOD1 Tg mice were analyzed as function of age in weeks. Factorial ANOVA did not discern an effect of treatment (p = 0.7551) or combined effect of treatment and age (p = 0.8662). (1.00 MB DOC) [file pone.0002740.s001.tif]

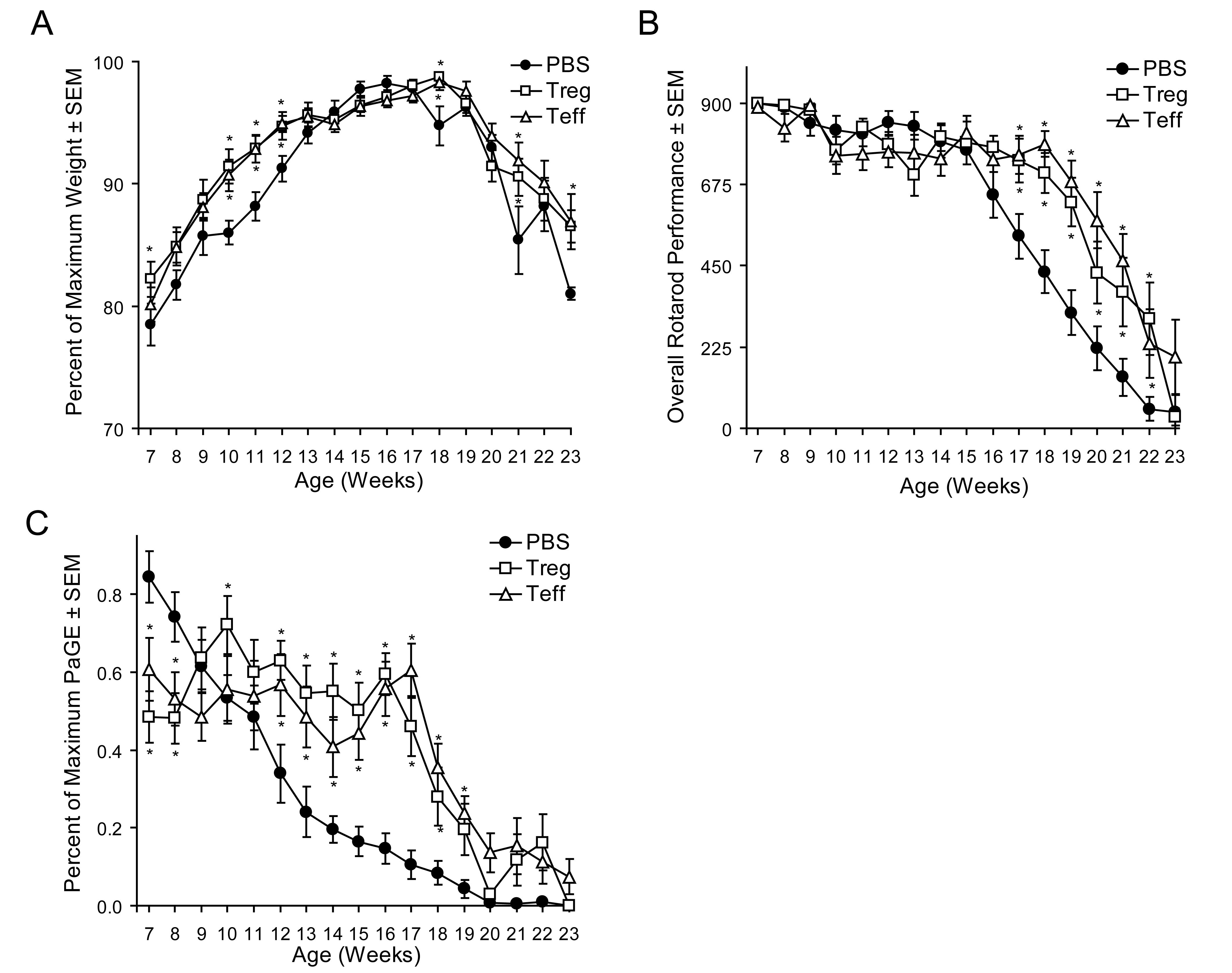

Supplement: Figure S2 — Adoptive transfer of anti-CD3 activated Wt Treg and Teff on weight and motor function of B6 G93A-SOD1 Tg mice. B6 G93A-SOD1 Tg mice (14–15 mice/group) were treated at 7, 13, and 19 weeks of age with PBS (closed circles), 1×10^6^ activated Treg (open boxes), or 1×10^6^ activated Teff (open triangles). (A) Mean body weights of treated SOD1 Tg mice were normalized to percentage of maximum weight±SEM and analyzed as function of age in weeks. Factorial ANOVA of percent maximum body weights indicated a significant combined effect of treatment and age (p = 0.0356). *P<0.05 compared to PBS-treated group at each time point by Fisher's LSD post-hoc tests. (B) Mean percentage of maximum overall rotarod performance (ORP)±SEM of treated SOD1 Tg mice analyzed as function of age in weeks. Factorial ANOVA indicated a significant combined effect of treatment and age (p = 3.3×10^−8^). *P<0.05 compared to PBS-treated group at each time point by Fisher's LSD post-hoc tests. (C) Mean percentage of maximum paw grip endurance (PaGE)±SEM of treated SOD1 Tg mice were analyzed as function of age in weeks. Factorial ANOVA indicated a significant combined effect of treatment and age (p = 6.7×10^−11^). *P<0.05 compared to PBS-treated group at each time point by Fisher's LSD post-hoc tests. (0.30 MB TIF) [file pone.0002740.s002.tif]
